# Establishment of cancer-associated fibroblasts-related subtypes and prognostic index for prostate cancer through single-cell and bulk RNA transcriptome

**DOI:** 10.1038/s41598-023-36125-0

**Published:** 2023-06-03

**Authors:** Youliang Qian, Dechao Feng, Jie Wang, Wuran Wei, Qiang Wei, Ping Han, Lu Yang

**Affiliations:** 1 Department of Urology, Institute of Urology, West China Hospital, Sichuan University, Guoxue Xiang #37, Chengdu, 610041 People’s Republic of China; 2 Department of Urology, Chengdu Second People’s Hospital, Chengdu, China

**Keywords:** Cancer, Cancer microenvironment, Tumour biomarkers

## Abstract

Current evidence indicate that cancer-associated fibroblasts (CAFs) play an important role in prostate cancer (PCa) development and progression. In this study, we identified CAF-related molecular subtypes and prognostic index for PCa patients undergoing radical prostatectomy through integrating single-cell and bulk RNA sequencing data. We completed analyses using software R 3.6.3 and its suitable packages. Through single-cell and bulk RNA sequencing analysis, NDRG2, TSPAN1, PTN, APOE, OR51E2, P4HB, STEAP1 and ABCC4 were used to construct molecular subtypes and CAF-related gene prognostic index (CRGPI). These genes could clearly divide the PCa patients into two subtypes in TCGA database and the BCR risk of subtype 1 was 13.27 times higher than that of subtype 2 with statistical significance. Similar results were observed in MSKCC2010 and GSE46602 cohorts. In addtion, the molucular subtypes were the independent risk factor of PCa patients. We orchestrated CRGPI based on the above genes and divided 430 PCa patients in TCGA database into high- and low- risk groups according to the median value of this score. We found that high-risk group had significant higher risk of BCR than low-risk group (HR: 5.45). For functional analysis, protein secretion was highly enriched in subtype 2 while snare interactions in vesicular transport was highly enriched in subtype 1. In terms of tumor heterogeneity and stemness, subtype 1 showd higher levels of TMB than subtype 2. In addition, subtype 1 had significant higher activated dendritic cell score than subtype 2. Based on eight CAF-related genes, we developed two prognostic subtypes and constructed a gene prognostic index, which could predict the prognosis of PCa patients very well.

## Introduction

Prostate cancer (PCa) is one of the most frequent cancers in males and is most prevalent in males 65 and older, with over 1.4 million new cases and 375,000 deaths expected globally in 2020^1–7^. In recent years, with the continuous economic and social development and the extension of the average life expectancy, the incidence of PCa was also increasing year by year^8,9^. Currently, the treatment approaches for PCa mainly contain radical prostatectomy, radical radiotherapy, androgen deprivation therapy and chemotherapy, which depends on the patient’s disease progression^4,9–12^. However, about one-fourth to one-half of patients will experience biochemical recurrence (BCR) after radical therapy, which means the return of measurable PSA^3,11,13–15^. BCR is a critical event in PCa progression. In some subgroups with specific clinical risk factors, patients with BCR are more likely to suffer clinical recurrence, metastasis and cancer-specific mortality^13,16^. Therefore, identification of marker signature to predict BCR in PCa patients is of great clinical significance.

Tumor microenvironment (TME) is the direct niche of the tumor and is composed of various types of cells in the metabolic environment^17^. Cancer-associated fibroblasts (CAFs) are the major components of TME stroma and have an important role in tumorigenesis, proliferation, progression and invasion^18–22^. CAFs are mainly transformed by intrinsic fibroblasts or stellate cells in tissues stimulated by growth factors. In addition, epithelial cells, endothelial cells, and myeloid-derived mesenchymal stem cells in tumor tissues can also differentiate into CAFs^18–21^. CAFs can secrete growth factors and cytokines to inhibit the function of immune cell, thereby promoting tumor progression, invasion and migration^18,20^. In addition, CAFs can synthesize and remodel the extracellular matrix, which forms the penetration barriers to prevent the penetration of the drug and the immune cells into the tumor tissue, thus reducing the effectiveness of the tumor therapy^19,21^. Several studies indicate that high density of CAFs is associated with poor BCR-free survival in PCa patients^23,24^. Therefore, an insight into the relationship between CAFs in PCa and tumor metastasis, proliferation, progression, and drug resistance is essential.

In our study, we constructed CAF-related gene prognostic index (CRGPI) based on 8 CAF-related genes identified by single-cell and bulk RNA transcriptome. Based on the non-negative matrix factorization (NMF) algorithm and the 8 identified CAF-related genes, we divided these patients into two subgroups and validated its reliability in several external datasets, which could predict the prognosis of PCa patients and better guide clinical application in the future.

## Methods

### Data preparation

In terms of single-cell RNA sequencing level, we downloaded 453 markers related to CAFs from the tumor immunotherapy gene expression resource (TIGER) database (http://tiger.canceromics.org/#/singleCellImmune^25^, which contains single-cell transcriptome data of 2,116,945 immune cells from 655 samples including PCa^26^. For bulk RNA sequencing level, we used the PCa gene matrix and clinical features in TCGA database from our pervious study^14^. CAF abundance was calculated using EPIC algorithm^27^ owing to its higher correlation than other immune algorithms^23,28^ and prognosis analysis was conducted. Weighted gene co-expression network analysis (WGCNA) was used to caculate the CAF-related genes in TCGA database. After intersection of CAF markers and CAF-related genes, we detected the final genes using Lasso regression analysis. Subsequently, we constructed risk score using coefficients in Lasso regression and TCGA subtypes using nonnegative matrix factorization (NMF) method based on these genes. CAF-related gene prognostic index (CRGPI) =  − 0.108359658017165*NDRG2-0.170784032731384*TSPAN1-0.0575930783435198*PTN + 0.084664542685909*APOE-0.0308048566779615*OR51E2-0.0450336720010116*P4HB + 0.0392873624467431*STEAP1-0.0148044294523877*ABCC4. In TCGA database, 430 samples with complete BCR information were used, and the p value was smaller than 0.05 using log-rank test for BCR-free survival. Two cohorts were used externally validated the prognostic values of risk score and TCGA subtypes, including GSE46602^29^ and MSKCC2010^30,31^. The clinical features of molecular subtypes were analyzed.

### Mutaion landscape and functional diferences between two subtypes

RNA-sequencing profiles, genetic mutation and corresponding clinical information for PCa were downloaded from the TCGA database (https://portal.gdc.com). The data of mutations were downloaded and visualized using the maftools package in R software. Differences of mutation frequency between two subtypes were also conducted. In terms of funcational analysis, gene set enrichment analysis (GSEA) was performed using “c2.cp.kegg.v7.4.symbols.gmt” and “h.all.v7.4.symbols.gmt” from the molecular signatures database^32,33^. Based on gene expression and subtypes, the minimum gene set was defined as 5 while the maximum gene set was 5000. Resampling was performed as 1000 times. P value of < 0.05 and a false discovery rate of < 0.05 were considered statistically significant.

### Tumor stemness and heterogeneity analyses

Tumor stemness indexes included differentially methylated probes-based stemness scores (DMPss), DNA methylation-based stemness scores (DNAss), enhancer elements/DNA methylation-based stemness scores (ENHss), epigenetically regulated DNA methylation-based stemness scores (EREG-METHss), epigenetically regulated RNA expression-based stemness scores (EREG.EXPss), RNA expression-based stemness scores (RNAss)^34^ and mRNAsi algorithm^35^. Tumor heterogeneity included homologous recombination deficiency (HRD), loss of heterozygosity (LOH), neoantigen (NEO), tumor ploidy, tumor purity, mutant-allele tumor heterogeneity (MATH), tumor mutation burden (TMB) and microsatellite instability (MSI)^36,37^. The results of above indicators were obtained from our previous study^38,39^. We compared the differences of two subtypes using the Wilcoxon rank sum test.

### Tumor immune microenvironmen (TME)

**Figure 1:**
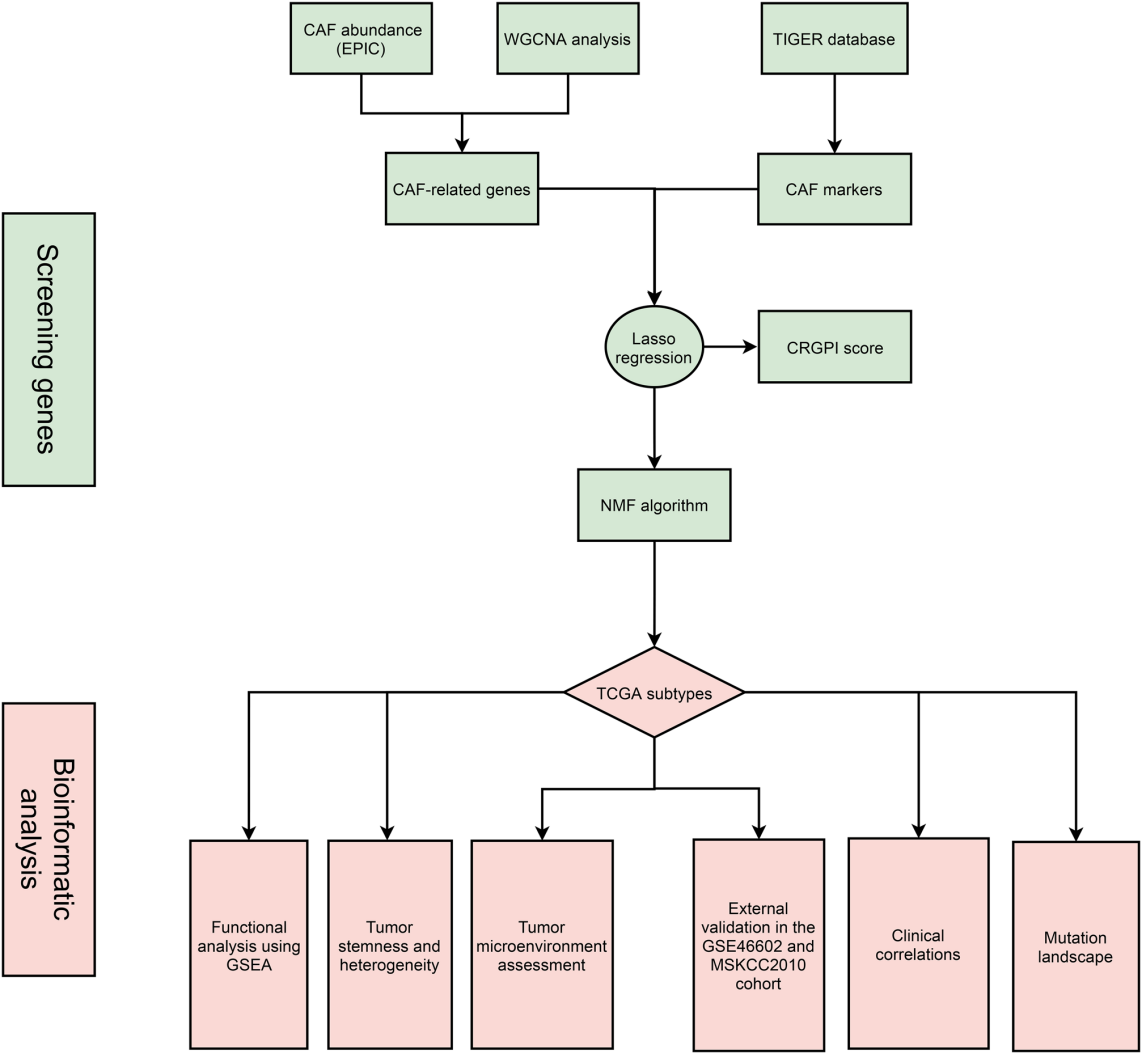
The flow chart of our study.

The overall tumor microenvironment and immune components assessment were calculated by Cibersort and ESTIMATE algorithms^40–42^. The differences of 54 immune checkpoints and tumor microenvironment scores betwenn the two subytpes were analyzed by the Wilcoxon rank sum test. Figure 1 illustrates the study flowchart.

### Statistical analysis

We completed analyses using software R 3.6.3 and its suitable packages. We exploited the Wilcoxon test in the context of abnormal distribution. Survival analysis was conducted through log-rank test and presented as Kaplan–Meier curve. Statistical significance was set as two-sided p < 0.05. Significant marks were as follows: not significance (ns), p ≥ 0.05; *p < 0.05; **p < 0.01; ***p < 0.001.

## Results

### Single cell and bulk RNA sequencing identified CAF-related markers and constructed TCGA subtypes and CRGPI

**Figure 2:**
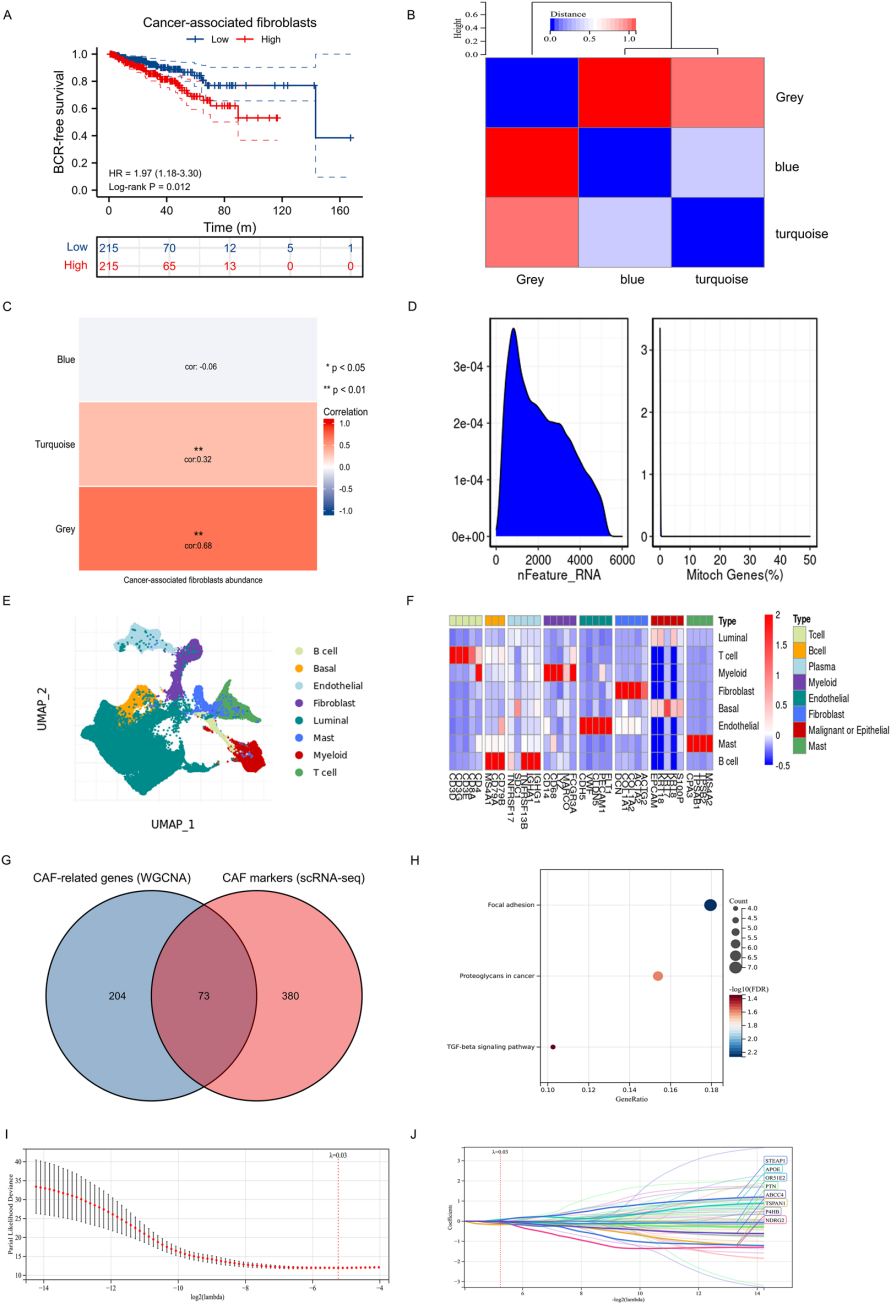
Identification of CAF-related genes. (**A**) prognostic difference between groups with high and low levels of CAF; (**B**) modules and genes through WGCNA analysis; (**C**) CAF-related genes through WGCNA analysis; (**D**) quality control of single-cell analysis; (**E**) cluster analysis of cells; (**F**) identification of fibroblasts; (**G**) Venn plot showing intersection of CAF-related genes and CAF markers; (**H**) KEGG analysis of intersected genes; (**I**) identifying optimal model using Lasso regression analysis; (**J**) results of Lasso regression analysis. *CAF* cancer-associated fibroblast, *WGCNA* weighted gene co-expression network analysis.

**Figure 3:**
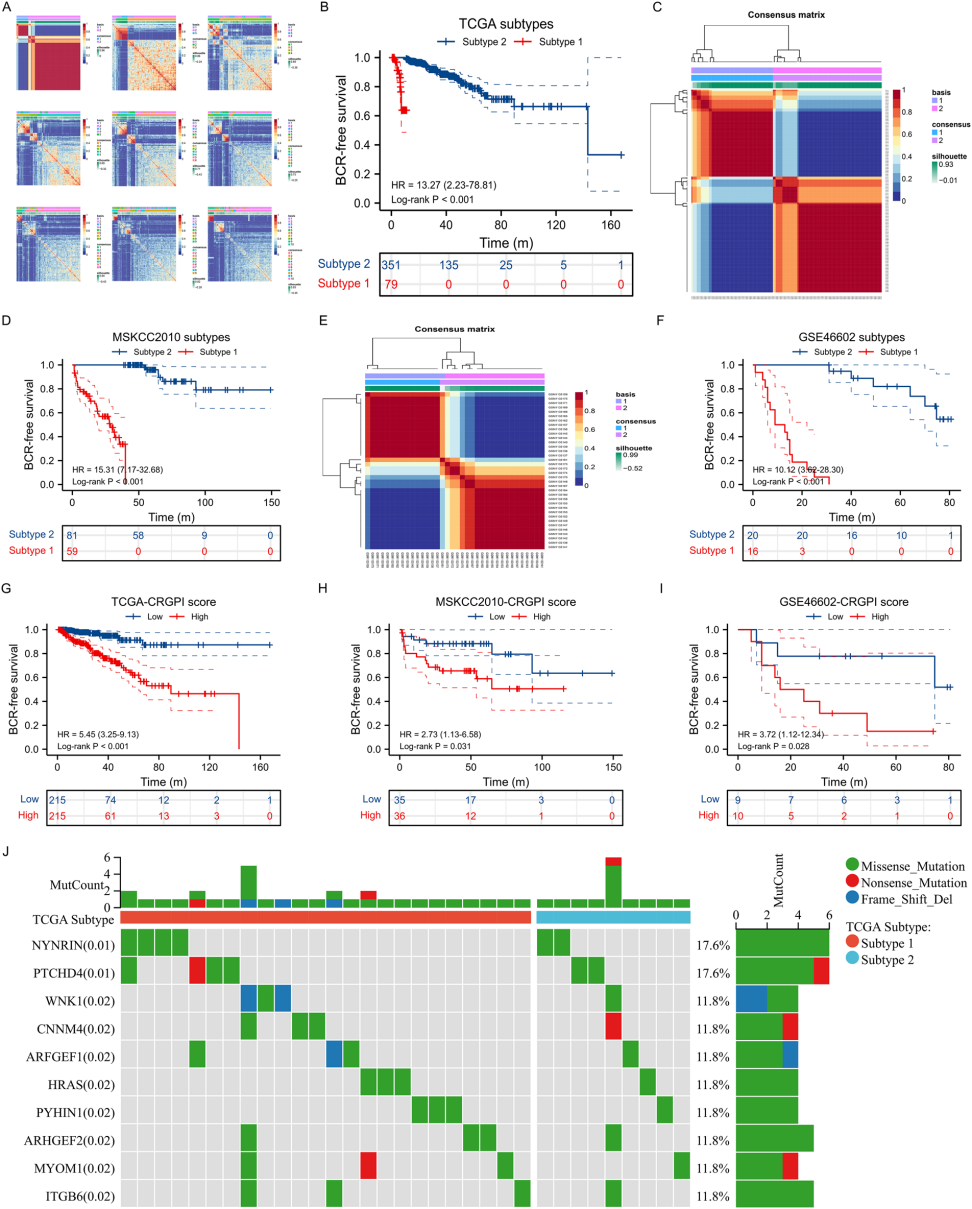
Identification of molecular subtypes and prognostic index. (**A**) heatmap showing all subtypes of prostate cancer patients in TCGA database; (**B**) Kaplan–Meier curve showing survival differences of subtype 1 and subtype 2 in TCGA database; (**C**) heatmap showing two subtypes of prostate cancer patients in MSKCC2010 cohort; (**D**) Kaplan–Meier curve showing survival differences of subtype 1 and subtype 2 in MSKCC2010 cohort; (**E**) heatmap showing two subtypes of prostate cancer patients in GSE46602 dataset; (**F**) Kaplan–Meier curve showing survival differences of subtype 1 and subtype 2 in GSE46602 dataset; (**G**) Kaplan–Meier curve showing survival differences of high- and low- CRGPI groups in TCGA database; (**H**) Kaplan–Meier curve showing survival differences of high- and low- CRGPI groups in MSKCC2010 cohort; (**I**) Kaplan–Meier curve showing survival differences of high- and low- CRGPI groups in GSE46602 dataset; (**J**) the mutation landscape between two subtypes. *CAF* cancer-associated fibroblast, *CRGPI* CAF-related gene prognostic index, *BCR* biochemical recurrence.

Using EPIC algorithm, we found that CAF was significantly associated with BCR-free survival in 430 PCa patients in TCGA database (Fig. 2A). Using WGCNA, three modules were found (Fig. 2B) where grey module genes was highly associated with CAF (Fig. 2C). In terms of single cell RNA sequencing data, Fig. 2D showed good quality control and tumor tissue consisted of eight types of cells, including fibroblasts (Fig. 2E), whose markers could be seen in Fig. 2F. Subsequently, we extracted 453 CAF markers using the definition of absolute value of logFC ≥ 0.4 and p value < 0.05 (Fig. 2D–F). In TCGA database, 277 genes were related to CAF using WGCNA. 73 genes were obtained from intersection of CAF-related genes and CAF markers (Fig. 2G) and these genes were involved in focal adhesion, proteoglycans in cancer and TCG-beta signaling pathway (Fig. 2H). Through Lasso regression analysis, when lambda (λ) equals 0.03, we obtained the optimal model (Fig. 2I) and used NDRG2, TSPAN1, PTN, APOE, OR51E2, P4HB, STEAP1 and ABCC4 for subsequent analysis (Fig. 2J). These genes could clearly divide the PCa patients into two subtypes in TCGA database (Fig. 3A) and the BCR risk of subtype 1 was 13.27 times higher than that of subtype 2 with statistical significance (Fig. 3B). Similar results were observed in MSKCC2010 (Fig. 3C,D) and GSE46602 cohorts (Fig. 3E,F). The baseline comparsion showed balanced clinical features between subtype 1 and subtype 2 (Table 1). In addtion, the molucular subtypes were the independent risk factor of PCa patients (Table 2). We orchestrated CRGPI based on the above genes and divided 430 PCa patients in TCGA database into high- and low- risk groups according to the median value of this score. We found that high-risk group had significant higher risk of BCR than low-risk group (HR: 5.45; Fig. 3G). Other two cohorts showed similar results (Fig. 3H,I). The top ten genes between subtype 1 and subtype 2 were NYNRIN, PTCHD4, WNK1, CNNM4, ARFGEF1, HRAS, PYHIN1, ARHGEF2, MYOM1 and ITGB6 with statistical significance (Fig. 3J).

**Table 1:** The clinical features between two subtypes in TCGA database.

Characteristics	Subtype 1	Subtype 2	P value
Sample	79	351	
Age, median (IQR)	62 (57, 66)	61 (56, 66)	0.647
Gleason score, n (%)			0.304
6	7 (8.86%)	32 (9.12%)	
7	33 (42.77%)	173 (49.29%)	
8	9 (11.39%)	50 (14.25%)	
9	30 (37.97%)	96 (27.35%)	
T stage, n (%)			1.000
T2	29 (37.18%)	126 (36.42%)	
T3	48 (61.54%)	213 (61.56%)	
T4	1 (1.28%)	7 (2.02%)	
Race, n (%)			0.234
Asian	2 (2.60%)	9 (2.65%)	
Black or African American	5 (6.49%)	45 (13.27%)	
White	70 (90.91%)	285 (84.07%)	
N stage, n (%)			0.897
N0	58 (82.86%)	248 (81.31%)	
N1	12 (17.14%)	57 (18.69%)	
Residual tumor, n (%)			0.659
No	48 (62.34%)	225 (65.79%)	
Yes	29 (37.66%)	117 (34.21%)	

*IQR* interquartile range.

**Table 2:** Cox analysis results including TCGA subtypes and clinical features.

Features	Total (N)	Univariate analysis		Multivariate analysis	
		Hazard ratio (95% CI)	P value	Hazard ratio (95% CI)	P value
TCGA subtype	430		** < 0.001**		
Subtype 2	351	Reference		Reference	
Subtype 1	79	108.183 (26.172–447.182)	** < 0.001**	187.874 (30.083–1173.332)	** < 0.001**
Age	430	1.016 (0.978–1.055)	0.426		
Gleason score	430		** < 0.001**		
GS6	39	Reference		Reference	
GS7	206	1.072 (0.242–4.757)	0.927	0.553 (0.068–4.494)	0.579
GS8-9	185	4.504 (1.090–18.611)	**0.038**	1.523 (0.189–12.281)	0.693
T stage	424		** < 0.001**		
T2	155	Reference		Reference	
T3	261	5.208 (2.230–12.163)	** < 0.001**	4.183 (1.540–11.362)	**0.005**
T4	8	6.140 (1.235–30.532)	**0.027**	4.412 (0.769–25.301)	0.096
Race	416		0.508		
White	355	Reference			
Asian	11	0.673 (0.147–3.079)	0.610		
Black or African American	50	0.648 (0.288–1.460)	0.296		
N stage	375		0.061		
N0	306	Reference		Reference	
N1	69	1.822 (1.001–3.313)	**0.049**	0.929 (0.493–1.748)	0.819
Residual tumor	419		**0.035**		
No	273	Reference		Reference	
Yes	146	1.781 (1.050–3.019)	**0.032**	1.105 (0.612–1.995)	0.741

*IQR* interquartile range.

Significant values are in bold.

### Functional enrichment, TME evaluation and tumor heterogeneity and stemness

**Figure 4:**
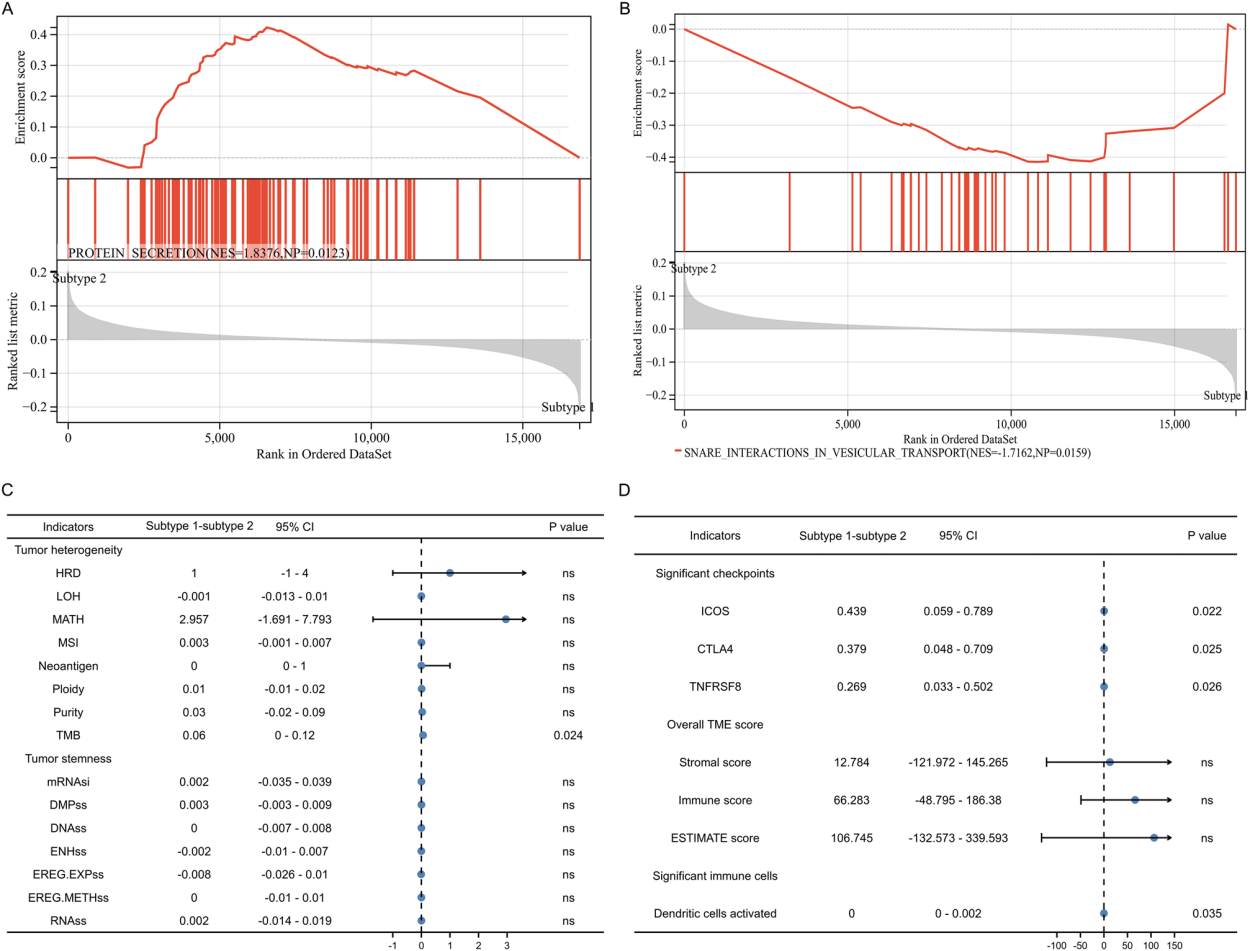
Differences between two subtypes in functional analysis, tumor heterogeneity and stemness, and immune landscape. (**A**,**B**) the functional differences between two subtypes; (**C**) the differences of tumor heterogeneity and tumor stemness between two subtypes; (**D**) the differences of tumor immune microenvironment scores and infiltrating cells between two subtypes. *TMB* tumor mutation burden, MATH mutant-allele tumor heterogeneity, *MSI* microsatellite instability, *NEO* neoantigen, *HRD* homologous recombination deficiency, *LOH* loss of heterozygosity, *DMPss* differentially methylated probes-based stemness scores, *DNAss* DNA methylation-based stemness scores, *ENHss* enhancer elements/DNA methylation-based stemness scores, *EREG*-*METHss* epigenetically regulated DNA methylation-based stemness scores, *EREG*.*EXPss* epigenetically regulated RNA expression-based stemness scores, *RNAss* RNA expression-based stemness scores.

For functional analysis, protein secretion was highly enriched in subtype 2 (Fig. 4A) while snare interactions in vesicular transport was highly enriched in subtype 1 (Fig. 4B). In terms of tumor heterogeneity and stemness, subtype 1 showd higher levels of TMB than subtype 2 (Fig. 4C). The expression levels of ICOS, CTLA4 and TNFRSF8 were significantly higher in subtype 1 than those in subtype 2 (Fig. 4D). In addition, subtype 1 had significant higher activated dendritic cell score than subtype 2 (Fig. 4D).

## Discussion

CAFs have been shown to promote tumor growth and progression in a variety of ways, such as secreting extracellular matrix proteins, inducing inflammation and neovascularization, increasing angiogenesis and constructing the immunosuppressive TME^18,43^. In PCa, CAFs are the most abundant cells in the stroma of TME^14^ and play an important role in the development and invasion of PCa^44,45^. For instance, CAFs can secrete IL-6 to enable AR transcriptional activity in PCa cells by modulating MAPK,STAT3,and PI3K/AKT signaling, thereby inducing resistance to anti-androgen therapies^44,46,47^. CAFs could mediate a series of genes such as NF-κB-dependent expression of the Wnt family member WNT16B to promote EMT in PCa cells, which induced resistance to cytotoxic agents^48^. Moreover, the role of CAFs in PCa bone metastasis has also been reported that CAFs may promote PCa bone metastasis by fibronectin and collagen deposition and establishing protein interaction network^49^. The important role of CAFs in tumorigenesis and development makes us aware that target CAFs will be an attractive breakthrough in anti-cancer therapy. In our study, we identified eight CAF-related genes by combining with single-cell and bulk RNA transcriptome, including STEAP1, APOE, OR51E2, PTN, ABCC4, TSPAN1, P4HB and NDRG2. Studies on the role of STEAP1, OR51E2, PTN, ABCC4, NDRG2 have been relatively in-depth. For instance, STEAP1 is all-called six-transmembrane epithelial antigen of the prostate 1, which belongs to a family of metalloproteinases involved in iron and copper homeostasis and other cellular processes^37^. STEAP1 is overexpressed on the plasma membrane of PCa cells and is associated with PCa invasiveness and metastasis^50^. The possible mechanism of STEAP1 promoting tumor proliferation and metastasis is by acting as a channel for small molecules that are involved in intercellular communication^50,51^. Similarly,OR51E2 is a kind of olfactory receptor, which is also called prostate specific G-protein receptor 2, and is highly expressed in PCa^52^. Some studies reported that OR51E2 perhaps could be used as one of the biomarkers for PCa^53–56^. Moreover, many researchers have shown that the activation of OR51E2 can inhibit proliferation of PCa cells and induce their invasion^57–60^. PTN encodes pleiotrophin, which is a secreted growth factor involved in angiogenesis and tumor growth^61^.A recent study showed that serum pleiotrophin levels were increased in the high-risk group compared with benign and low-risk PCa patients^62^, which is consistent with our previous study^63^. The protein encoded by ABCC4 is a member of the superfamily of ATP-binding cassette (ABC) transporters, which is also called multidrug resistance protein 4 (MRP4)^64^. MRP4 was reported to be associated with drug resistance of PCa in many studies^65–67^. NDRG2 is a tumor suppressor gene that suppresses tumorigenesis and metastasis and increases the sensitivity to anticancer drugs^68^. Several researches have reported that the overexpression of NDRG2 could suppress the invasion and metastasis of PCa cells^69,70^. Some researchers found that low level of NDRG2 were associated with radioresistance of PCa Cells and overexpression of NDRG2 in combination with radiotherapy might be an effective therapeutic method for PCa^71,72^. In summary, the function of these five genes in PCa is relatively determined. There are very few reports of TSPAN1 and P4HB in PCa. The overexpression of TSPAN1 is widely regarded as with EMT, tumor proliferation and migration, tumor growth which has been demonstrated in multiple cancer types^73–77^. In PCa, TSPAN1 was induced by androgens and upregulated in PCa tissues. TSPAN1 involved in controlling the expression of key proteins of cell migration to promote invasion and metastasis of PCa^78^. In addition, another study found that the knockdown of TSPAN1 in PCa cell could inhibit cell proliferation and migration^79^. However, the detailed mechanisms of regulating the proliferation and metastasis of PCa cells remain incompletely elucidated and require further investigation. There is only one study on the role of P4HB in PCa. Hu et al. constructed an autophagy-related gene signature containing P4HB and other genes in PCa through bioinformatic analysis^80^. But there are many studies on the role of P4HB in other diseases, such as bladder carcinoma, oesophageal cancer and kidney renal clear cell carcinoma^81–83^. There seems to be some debates about the role of APOE in PCa. A study showed that different apolipoprotein E genotypes may have different risks of developing PCa^84^. However, another study showed apolipoprotein E genotype was not associated with PCa risk^85^. Accurate conclusions may need to be supported by more experimental evidence. In summary, based on the eight identified genes, we divided 430 PCa patients into 2 subtypes. We observed the prognosis of subgroup 1 was significantly worse than that of subgroup 2. To better reveal the potential mechanisms of the two prognostic subtypes, we performed functional analysis, gene mutation, tumor heterogeneity and stemness, and TME assessment.

Our study found protein secretion was highly enriched in subtype 2 while snare interactions in vesicular transport was highly enriched in subtype 1.Snare is all-called soluble *N*-ethylmaleimide-sensitive factor attachment protein receptors, which plays an important role in tumor invasion, chemo-resistance, autophagy, apoptosis, and phosphorylation of kinases essential for cancer cell biogenesis^86^. Snare can promote close proximity between vesicles and cell membrane to form a fusion pore after contact, which can mediate the transport of material between vesicles and cells^86,87^. Therefore, the enrichment of snare interactions in vesicular transport pathway in cluster 1 may indicate a higher level of proliferation and invasion demand for PCa cells in cluster 1, correlating with a worse prognosis. In contrast, we observed that protein secretion was mainly enriched in subtype 2 and we speculated it may be related to the increasing androgen signaling. Compared with hormone-naive metastatic PCa, hormone-refractory metastatic PCa showed a marked decrease of androgen signaling and protein biosynthesis, which possibly meant the decrease of androgen signaling and protein biosynthesis was responsible for PCa progression and higher malignancy of tumor cells^88^. This is agreement with our study showing patients in subtype 2 have better prognosis. Additionally, the difference of TMB between subtype 1 and subtype 2 may also explain why the prognosis of subtype 1 is much worse than subtype 2. We observed TMB of subtype 1 was significantly higher than subtype 2. TMB refers to the total number of mutations present in a single tumor specimen, which can be used to predict the tumor response to immunotherapy in a variety of tumors^89^. Patients with high TMB usually mean that tumor cells carry more tumor antigens on their surface, which are therefore vulnerable to being killed by activated immune cells^89,90^. In PCa, high TMB level was significantly associated with older age, positive lymph node, higher international Society of Urological Pathology (ISUP) grade, advanced stage and poor BCR-free survival^91^. In metastatic castration-resistant PCa, ICIs were more effective than taxanes for patients with high TMB(10 mt/Mb or greater)^92^. These two studies showed PCa patients with higher TMB were associated with bad prognosis and better effect of immunotherapy, which was consistent with our study and may explain the poor prognosis of subgroup 1 to some extent. Furthermore, we compared the differences in the common immune checkpoints between subtype 1 and 2 and found the expression of ICOS, CTLA4 and TNFRSF8 were significantly elevated in subtype 1.It is well known that a significant mechanism of tumor cells evading immunosurveillance is activation of immune checkpoint pathways, which can suppress antitumor immune response^93^. CTLA-4 has been widely studied in tumors. High expression of CTLA-4 was observed to be associated with a worse prognosis in many cancers, including PCa, and the intervention of CTLA-4 blockers could improve prognosis of patients^94–98^. High expression of CTLA-4 can inhibit T cell activation by competing with CD28, regulating the inhibitory function of Treg cells and controlling adhesion and motility, which in turn leads to immunosuppression of tumors. Similarly, ICOS is an activating costimulatory immune checkpoint expressed on activated T cells, which participate in regulating T cell activation and adaptive immune responses. Mo L et al. adopt a combination approach of ICOS positive Treg cells depletion with tumor cell vaccine in mouse PCa model and found ICOS blocking could deplete the tumor-infiltrated ICOS positive Treg cells^99^. These findings may well explain our results, because high expression of CTLA-4 and ICOS may mean stronger immunosuppression, which then leads to a worse prognosis of the subtype 1 patients. Moreover,TNFRSF8 is an important therapeutic target for the treatment of malignant lymphomas, but there are few studies on TNFRSF8 in nonlymphoid tumors^100^. Only a recent study showed higher plasma TNFRSF8 were associated with shorter PFS in patients received androgen deprivation therapy plus cabozantinib^101^. More evidence is needed regarding whether the high expression of TNFRSF8 is associated with poor prognosis.

Intriguingly, we observed in the comparison of dendritic cells activated, subtype 1 was lower than subtype 2. The median difference between the two groups was 0 (0–0.002), and the difference was statistically significant. There are already many evidences that dendritic cells plays an important role in antitumor immunity^102^. Dendritic cells can capture tumor antigens that are released from tumor cells and present them to T cells, eventually resulting in the generation of cytotoxic T lymphocytes (CTLs)^103^. This may indicate that with increasing dendritic cells activation, the antigen presentation function is increasing, and then the number and function of CTLs is increasing, ultimately leading to a stronger antitumor immune response. In addition, the success of Sipuleucel-T (a dendritic cell based immunotherapy) in prostate tumor therapy reflects the great value of immunotherapy targeting dendritic cells^104^. In PCa, there have been a phase 3 trial showing that Sipuleucel-T significantly improve overall survival in patients with metastatic castration-resistant PCa^105^.These studies are consistent with our results and reveal the important role of dendritic cells activated in the prognosis of patients with PCa.

## Conclusions

Based on eight CAF-related genes, we developed two prognostic subtypes and constructed a gene prognostic index, which could predict the prognosis of PCa patients very well. Meanwhile, our study also provides a valuable resource for understanding the underlying mechanisms of PCa progression, and provides valuable insights into the management of the BCR in PCa.

## Data Availability

The datasets generated and/or analyzed during the current study are available in the TCGA (https://www.cancer.gov/tcga) and GEO (https://www.ncbi.nlm.nih.gov/geo/, accession number: GSE116918 and GSE46602) repositories.

## References

[1] Sung H, Ferlay J, Siegel RL, Laversanne M, Soerjomataram I, Jemal A (2021). Global cancer statistics 2020: GLOBOCAN estimates of incidence and mortality worldwide for 36 cancers in 185 countries. CA.

[2] Feng D, Li D, Shi X, Xiong Q, Zhang F, Wei Q (2022). A gene prognostic index from cellular senescence predicting metastasis and radioresistance for prostate cancer. J. Transl. Med..

[3] Feng D, Shi X, Xiong Q, Zhang F, Li D, Yang L (2021). A gene prognostic index associated with epithelial-mesenchymal transition predicting biochemical recurrence and tumor chemoresistance for prostate cancer. Front. Oncol..

[4] Feng D, Shi X, Zhang F, Xiong Q, Wei Q, Yang L (2022). Mitochondria dysfunction-mediated molecular subtypes and gene prognostic index for prostate cancer patients undergoing radical prostatectomy or radiotherapy. Front. Oncol..

[5] Weitao Zheng DF, Xiong X, Liao X, Wang S, Hang X, Le W, Wei Q, Yang L (2023). The role of cGAS-STING in age-related diseases from mechanisms to therapies. Aging Dis..

[6] Megerian MF, Kim JS, Badreddine J, Hong SH, Ponsky LE, Shin JI, Ghayda RA (2022). Melatonin and prostate cancer: Anti-tumor roles and therapeutic application. Aging Dis..

[7] Zhengshuai Song Q.C., Bin Guo, Ye Zhao, Xuechao Li, Ning Lou, Chenxi Zhu, Gang Luo, Song Peng, Guohao Li, Ke Chen, Yong Wang, Hailong Ruan, Yonglian Guo. Overexpression of RACGAP1 by E2F1 Promotes Neuroendocrine Differentiation of Prostate Cancer by Stabilizing EZH2 Expression. Aging and Disease (2023). 10.14336/AD.2023.0202 PMC10529746 37196108

[8] Daniyal M, Siddiqui ZA, Akram M, Asif HM, Sultana S, Khan A (2014). Epidemiology, etiology, diagnosis and treatment of prostate cancer. Asian Pac. J. Cancer Prev..

[9] Feng D, Xiong Q, Zhang F, Shi X, Xu H, Wei W (2022). Identification of a novel nomogram to predict progression based on the circadian clock and insights into the tumor immune microenvironment in prostate cancer. Front. Immunol..

[10] Litwin MS, Tan H-J (2017). The diagnosis and treatment of prostate cancer: A review. JAMA.

[11] Feng D, Shi X, Xiong Q, Zhang F, Li D, Wei W (2022). A Ferroptosis-related gene prognostic index associated with biochemical recurrence and radiation resistance for patients with prostate cancer undergoing radical radiotherapy. Front. Cell Dev. Biol..

[12] Feng D, Zhang F, Li D, Shi X, Xiong Q, Wei Q (2022). Developing an immune-related gene prognostic index associated with progression and providing new insights into the tumor immune microenvironment of prostate cancer. Immunology.

[13] Van den Broeck T, van den Bergh RCN, Arfi N, Gross T, Moris L, Briers E (2019). Prognostic value of biochemical recurrence following treatment with curative intent for prostate cancer: A systematic review. Eur. Urol..

[14] Feng D, Shi X, Zhang F, Xiong Q, Wei Q, Yang L (2022). Energy metabolism-related gene prognostic index predicts biochemical recurrence for patients with prostate cancer undergoing radical prostatectomy. Front. Immunol..

[15] Feng D, Zhu W, Shi X, Wang Z, Wei W, Wei Q (2023). Immune-related gene index predicts metastasis for prostate cancer patients undergoing radical radiotherapy. Exp. Hematol. Oncol..

[16] Roobol MJ, Carlsson SV (2013). Risk stratification in prostate cancer screening. Nat. Rev. Urol..

[17] Xing S, Hu K, Wang Y (2022). Tumor immune microenvironment and immunotherapy in non-small cell lung cancer: Update and new challenges. Aging Dis..

[18] Sahai E, Astsaturov I, Cukierman E, DeNardo DG, Egeblad M, Evans RM (2020). A framework for advancing our understanding of cancer-associated fibroblasts. Nat. Rev. Cancer.

[19] Räsänen K, Vaheri A (2010). Activation of fibroblasts in cancer stroma. Exp. Cell Res..

[20] Nakagawa H, Liyanarachchi S, Davuluri RV, Auer H, Martin EW, de la Chapelle A (2004). Role of cancer-associated stromal fibroblasts in metastatic colon cancer to the liver and their expression profiles. Oncogene.

[21] Kalluri R (2016). The biology and function of fibroblasts in cancer. Nat. Rev. Cancer.

[22] Yang Y, Gu J, Li X, Xue C, Ba L, Gao Y (2021). HIF-1alpha promotes the migration and invasion of cancer-associated fibroblasts by miR-210. Aging Dis..

[23] Feng D, Xiong Q, Wei Q, Yang L (2022). Cellular landscape of tumour microenvironment in prostate cancer. Immunology.

[24] Wu Z, Shi J, Lai C, Li K, Li K, Li Z (2021). Clinicopathological significance and prognostic value of cancer-associated fibroblasts in prostate cancer patients. Urol. Oncol..

[25] Chen Z, Luo Z, Zhang D, Li H, Liu X, Zhu K (2022). TIGER: A web portal of tumor immunotherapy gene expression resource. Genom. Proteom. Bioinform..

[26] Chen S, Zhu G, Yang Y, Wang F, Xiao YT, Zhang N (2021). Single-cell analysis reveals transcriptomic remodellings in distinct cell types that contribute to human prostate cancer progression. Nat. Cell Biol..

[27] Racle J, de Jonge K, Baumgaertner P, Speiser DE, Gfeller D (2017). Simultaneous enumeration of cancer and immune cell types from bulk tumor gene expression data. Elife.

[28] Sturm G, Finotello F, Petitprez F, Zhang JD, Baumbach J, Fridman WH (2019). Comprehensive evaluation of transcriptome-based cell-type quantification methods for immuno-oncology. Bioinformatics.

[29] Mortensen MM, Hoyer S, Lynnerup AS, Orntoft TF, Sorensen KD, Borre M (2015). Expression profiling of prostate cancer tissue delineates genes associated with recurrence after prostatectomy. Sci. Rep..

[30] Cerami E, Gao J, Dogrusoz U, Gross BE, Sumer SO, Aksoy BA (2012). The cBio cancer genomics portal: An open platform for exploring multidimensional cancer genomics data. Cancer Discov..

[31] Gao J, Aksoy BA, Dogrusoz U, Dresdner G, Gross B, Sumer SO (2013). Integrative analysis of complex cancer genomics and clinical profiles using the cBioPortal. Sci. Signal..

[32] Liberzon A, Subramanian A, Pinchback R, Thorvaldsdottir H, Tamayo P, Mesirov JP (2011). Molecular signatures database (MSigDB) 3.0. Bioinformatics.

[33] Subramanian A, Tamayo P, Mootha VK, Mukherjee S, Ebert BL, Gillette MA (2005). Gene set enrichment analysis: A knowledge-based approach for interpreting genome-wide expression profiles. Proc. Natl. Acad. Sci. U. S. A..

[34] Huang TX, Fu L (2019). The immune landscape of esophageal cancer. Cancer Commun. (Lond)..

[35] Malta TM, Sokolov A, Gentles AJ, Burzykowski T, Poisson L, Weinstein JN (2018). machine learning identifies stemness features associated with oncogenic dedifferentiation. Cell.

[36] Bonneville R, Krook MA, Kautto EA, Miya J, Wing MR, Chen HZ (2017). Landscape of microsatellite instability across 39 cancer types. JCO Precis. Oncol..

[37] Thorsson V, Gibbs DL, Brown SD, Wolf D, Bortone DS, Ou Yang TH (2018). The immune landscape of cancer. Immunity.

[38] Feng D, Shi X, Zhu W, Zhang F, Li D, Han P (2022). A pan-cancer analysis of the oncogenic role of leucine zipper protein 2 in human cancer. Exp. Hematol. Oncol..

[39] Zhu W, Feng D, Shi X, Li D, Wei Q, Yang L (2022). A pan-cancer analysis of the oncogenic role of zinc finger protein 419 in human cancer. Front. Oncol..

[40] Yoshihara K, Shahmoradgoli M, Martinez E, Vegesna R, Kim H, Torres-Garcia W (2013). Inferring tumour purity and stromal and immune cell admixture from expression data. Nat. Commun..

[41] Zeng D, Ye Z, Shen R, Yu G, Wu J, Xiong Y (2021). IOBR: Multi-omics immuno-oncology biological research to decode tumor microenvironment and signatures. Front. Immunol..

[42] Newman AM, Liu CL, Green MR, Gentles AJ, Feng W, Xu Y (2015). Robust enumeration of cell subsets from tissue expression profiles. Nat. Methods.

[43] Biffi G, Tuveson DA (2021). Diversity and biology of cancer-associated fibroblasts. Physiol. Rev..

[44] Bonollo F, Thalmann GN, Kruithof-de Julio M, Karkampouna S (2020). The role of cancer-associated fibroblasts in prostate cancer tumorigenesis. Cancers.

[45] Chiarugi P, Paoli P, Cirri P (2014). Tumor microenvironment and metabolism in prostate cancer. Semin. Oncol..

[46] Ishii K, Sasaki T, Iguchi K, Kajiwara S, Kato M, Kanda H (2018). Interleukin-6 induces VEGF secretion from prostate cancer cells in a manner independent of androgen receptor activation. Prostate.

[47] Yang L, Wang L, Lin H-K, Kan P-Y, Xie S, Tsai M-Y (2003). Interleukin-6 differentially regulates androgen receptor transactivation via PI3K-Akt, STAT3, and MAPK, three distinct signal pathways in prostate cancer cells. Biochem. Biophys. Res. Commun..

[48] Sun Y, Campisi J, Higano C, Beer TM, Porter P, Coleman I (2012). Treatment-induced damage to the tumor microenvironment promotes prostate cancer therapy resistance through WNT16B. Nat. Med..

[49] Özdemir BC, Hensel J, Secondini C, Wetterwald A, Schwaninger R, Fleischmann A (2014). The molecular signature of the stroma response in prostate cancer-induced osteoblastic bone metastasis highlights expansion of hematopoietic and prostate epithelial stem cell niches. PLoS One.

[50] Xu M, Evans L, Bizzaro CL, Quaglia F, Verrillo CE, Li L (2022). STEAP1–4 (six-transmembrane epithelial antigen of the prostate 1–4) and their clinical implications for prostate cancer. Cancers.

[51] Challita-Eid PM, Morrison K, Etessami S, An Z, Morrison KJ, Perez-Villar JJ (2007). Monoclonal antibodies to six-transmembrane epithelial antigen of the prostate-1 inhibit intercellular communication in vitro and growth of human tumor xenografts in vivo. Can. Res..

[52] Abaffy T, Bain JR, Muehlbauer MJ, Spasojevic I, Lodha S, Bruguera E (2018). A testosterone metabolite 19-hydroxyandrostenedione induces neuroendocrine trans-differentiation of prostate cancer cells via an ectopic olfactory receptor. Front. Oncol..

[53] Rodriguez M, Siwko S, Liu M (2016). Prostate-specific G-protein coupled receptor, an emerging biomarker regulating inflammation and prostate cancer invasion. Curr. Mol. Med..

[54] Souza MFd, Kuasne H, Barros-Filho MdC, Cilião HL, Marchi FA, Fuganti PE (2017). Circulating mRNAs and miRNAs as candidate markers for the diagnosis and prognosis of prostate cancer. Plos One.

[55] Rigau M, Morote J, Mir MC, Ballesteros C, Ortega I, Sanchez A (2010). PSGR and PCA3 as biomarkers for the detection of prostate cancer in urine: Urine assay for the detection of prostate cancer. Prostate.

[56] Wang J, Weng J, Cai Y, Penland R, Liu M, Ittmann M (2006). The prostate-specific G-protein coupled receptors PSGR and PSGR2 are prostate cancer biomarkers that are complementary to α-methylacyl-CoA racemase. Prostate.

[57] Pronin A, Slepak V (2021). Ectopically expressed olfactory receptors OR51E1 and OR51E2 suppress proliferation and promote cell death in a prostate cancer cell line. J. Biol. Chem..

[58] Li Y, Li Q, Li D, Gu J, Qian D, Qin X (2021). Exosome carrying PSGR promotes stemness and epithelial-mesenchymal transition of low aggressive prostate cancer cells. Life Sci..

[59] Jovancevic N, Khalfaoui S, Weinrich M, Weidinger D, Simon A, Kalbe B (2017). Odorant receptor 51E2 agonist β-ionone regulates RPE cell migration and proliferation. Front. Physiol..

[60] Neuhaus EM, Zhang W, Gelis L, Deng Y, Noldus J, Hatt H (2009). Activation of an olfactory receptor inhibits proliferation of prostate cancer cells. J. Biol. Chem..

[61] Polytarchou C, Hatziapostolou M, Poimenidi E, Mikelis C, Papadopoulou A, Parthymou A (2009). Nitric oxide stimulates migration of human endothelial and prostate cancer cells through up-regulation of pleiotrophin expression and its receptor protein tyrosine phosphatase β/ζ. Int. J. Cancer.

[62] Liu S, Shen M, Hsu E-C, Zhang CA, Garcia-Marques F, Nolley R (2021). Discovery of PTN as a serum-based biomarker of pro-metastatic prostate cancer. Br. J. Cancer.

[63] Feng DC, Zhu WZ, Shi X, Xiong Q, You J, Wei Q (2022). Identification of senescence-related molecular subtypes and key genes for prostate cancer. Asian J. Androl..

[64] Ritter CA, Jedlitschky G, Meyer zu Schwabedissen H, Grube M, Köck K, Kroemer HK (2005). Cellular export of drugs and signaling molecules by the ATP-binding cassette transporters MRP4 (ABCC4) and MRP5 (ABCC5). Drug Metab. Rev..

[65] Oprea-Lager DE, Bijnsdorp IV, Van Moorselaar RJA, Van Den Eertwegh AJM, Hoekstra OS, Geldof AA (2013). ABCC4 Decreases docetaxel and not cabazitaxel efficacy in prostate cancer cells in vitro. Anticancer Res..

[66] Huang H, Li J, Shen J, Lin L, Wu X, Xiang S (2020). Increased ABCC4 expression induced by ERRα leads to docetaxel resistance via efflux of docetaxel in prostate cancer. Front. Oncol..

[67] Ho LL, Kench JG, Handelsman DJ, Scheffer GL, Stricker PD, Grygiel JG (2008). Androgen regulation of multidrug resistance-associated protein 4 (MRP4/ABCC4) in prostate cancer. Prostate.

[68] Lee KW, Lim S, Kim KD (2022). The function of N-Myc downstream-regulated gene 2 (NDRG2) as a negative regulator in tumor cell metastasis. Int. J. Mol. Sci..

[69] Gao L, Wu G-J, Liu X-W, Zhang R, Yu L, Zhang G (2011). Suppression of invasion and metastasis of prostate cancer cells by overexpression of NDRG2 gene. Cancer Lett..

[70] Moradi Monfared M, Alizadeh Zarei M, Rafiei Dehbidi G, Behzad Behbahani A, Arabsolghar R, Takhshid MA (2019). NDRG2 regulates the expression of genes involved in epithelial mesenchymal transition of prostate cancer cells. Iran. J. Med. Sci..

[71] Wang W, Liu M, Guan Y, Wu Q (2016). Hypoxia-responsive Mir-301a and Mir-301b promote radioresistance of prostate cancer cells via downregulating NDRG2. Med. Sci. Monit..

[72] Alizadeh Zarei M, Takhshid MA, Behzad Behbahani A, Hosseini SY, Okhovat MA, Rafiee Dehbidi GR (2017). Synergistic effects of NDRG2 overexpression and radiotherapy on cell death of human prostate LNCaP cells. J. Biomed. Phys. Eng..

[73] Garcia-Mayea Y, Mir C, Carballo L, Sánchez-García A, Bataller M, LLeonart ME (2022). TSPAN1, a novel tetraspanin member highly involved in carcinogenesis and chemoresistance. Biochim. Biophys. Acta Rev. Cancer.

[74] Wang Y, Liang Y, Yang G, Lan Y, Han J, Wang J (2018). Tetraspanin 1 promotes epithelial-to-mesenchymal transition and metastasis of cholangiocarcinoma via PI3K/AKT signaling. J. Exp. Clin. Cancer Res..

[75] Zhang X, Shi G, Gao F, Liu P, Wang H, Tan X (2019). TSPAN1 upregulates MMP2 to promote pancreatic cancer cell migration and invasion via PLCγ. Oncol. Rep..

[76] Wang G-L, Chen L, Wei Y-Z, Zhou J-M, Wu Y-Y, Zhang Y-X (2012). The effect of NET-1 on the proliferation, migration and endocytosis of the SMMC-7721 HCC cell line. Oncol. Rep..

[77] Garcia-Mayea Y, Mir C, Carballo L, Castellvi J, Temprana-Salvador J, Lorente J (2020). TSPAN1: A novel protein involved in head and neck squamous cell carcinoma chemoresistance. Cancers.

[78] Munkley J, McClurg UL, Livermore KE, Ehrmann I, Knight B, Mccullagh P (2017). The cancer-associated cell migration protein TSPAN1 is under control of androgens and its upregulation increases prostate cancer cell migration. Sci. Rep..

[79] Xu F, Gao Y, Wang Y, Pan J, Sha J, Shao X (2016). Decreased TSPAN1 promotes prostate cancer progression and is a marker for early biochemical recurrence after radical prostatectomy. Oncotarget.

[80] Hu D, Jiang L, Luo S, Zhao X, Hu H, Zhao G (2020). Development of an autophagy-related gene expression signature for prognosis prediction in prostate cancer patients. J. Transl. Med..

[81] Wu Y, Peng Y, Guan B, He A, Yang K, He S (2021). P4HB: A novel diagnostic and prognostic biomarker for bladder carcinoma. Oncol. Lett..

[82] Gao X, Wang Y, Lu F, Chen X, Yang D, Cao Y (2021). Extracellular vesicles derived from oesophageal cancer containing P4HB promote muscle wasting via regulating PHGDH/Bcl-2/caspase-3 pathway. J. Extracell.r Vesicles.

[83] Xie L, Li H, Zhang L, Ma X, Dang Y, Guo J (2020). Autophagy-related gene P4HB: A novel diagnosis and prognosis marker for kidney renal clear cell carcinoma. Aging.

[84] Yencilek F, Yilmaz SG, Yildirim A, Gormus U, Altinkilic EM, Dalan AB (2016). Apolipoprotein E genotypes in patients with prostate cancer. Anticancer Res..

[85] Liu H, Shui IM, Platz EA, Mucci LA, Giovannucci EL (2015). No association of apoe genotype with risk of prostate cancer: A nested case-control study. Cancer Epidemiol. Biomark. Prev..

[86] Meng J, Wang J (2015). Role of SNARE proteins in tumourigenesis and their potential as targets for novel anti-cancer therapeutics. Biochem. Biophys. Acta.

[87] Söllner TH, Rothman JE (1996). Molecular machinery mediating vesicle budding, docking and fusion. Cell Struct. Funct..

[88] Tomlins SA, Mehra R, Rhodes DR, Cao X, Wang L, Dhanasekaran SM (2007). Integrative molecular concept modeling of prostate cancer progression. Nat. Genet..

[89] Chan TA, Yarchoan M, Jaffee E, Swanton C, Quezada SA, Stenzinger A (2019). Development of tumor mutation burden as an immunotherapy biomarker: Utility for the oncology clinic. Ann. Oncol..

[90] Jardim DL, Goodman A, de Melo GD, Kurzrock R (2021). The challenges of tumor mutational burden as an immunotherapy biomarker. Cancer Cell.

[91] Luo C, Chen J, Chen L (2020). Exploration of gene expression profiles and immune microenvironment between high and low tumor mutation burden groups in prostate cancer. Int. Immunopharmacol..

[92] Graf RP, Fisher V, Weberpals J, Gjoerup O, Tierno MB, Huang RSP (2022). Comparative effectiveness of immune checkpoint inhibitors vs chemotherapy by tumor mutational burden in metastatic castration-resistant prostate cancer. JAMA Netw. Open.

[93] Darvin P, Toor SM, Sasidharan Nair V, Elkord E (2018). Immune checkpoint inhibitors: Recent progress and potential biomarkers. Exp. Mol. Med..

[94] Kern R, Panis C (2021). CTLA-4 expression and its clinical significance in breast cancer. Arch. Immunol. Ther. Exp..

[95] Larkin J, Chiarion-Sileni V, Gonzalez R, Grob JJ, Cowey CL, Lao CD (2015). Combined nivolumab and ipilimumab or monotherapy in untreated melanoma. N. Engl. J. Med..

[96] Liu J-N, Kong X-S, Huang T, Wang R, Li W, Chen Q-F (2020). Clinical implications of aberrant PD-1 and CTLA4 expression for cancer immunity and prognosis: A pan-cancer study. Front. Immunol..

[97] Liang J, Hong J, Tang X, Qiu X, Zhu K, Zhou L (2022). Sex difference in response to non-small cell lung cancer immunotherapy: An updated meta-analysis. Ann. Med..

[98] Carosella ED, Ploussard G, LeMaoult J, Desgrandchamps F (2015). A systematic review of immunotherapy in urologic cancer: Evolving roles for targeting of CTLA-4, PD-1/PD-L1, and HLA-G. Eur. Urol..

[99] Mo L, Chen Q, Zhang X, Shi X, Wei L, Zheng D (2017). Depletion of regulatory T cells by anti-ICOS antibody enhances anti-tumor immunity of tumor cell vaccine in prostate cancer. Vaccine.

[100] Kumar A, Younes A (2014). Role of CD30 targeting in malignant lymphoma. Curr. Treat. Options Oncol..

[101] Corn PG, Zhang M, Nogueras-Gonzalez GM, Xiao L, Zurita AJ, Subudhi SK (2020). A phase II study of cabozantinib and androgen ablation in patients with hormone-naïve metastatic prostate cancer. Clin. Cancer Res..

[102] Anguille S, Smits EL, Lion E, van Tendeloo VF, Berneman ZN (2014). Clinical use of dendritic cells for cancer therapy. Lancet Oncol..

[103] Palucka K, Banchereau J (2012). Cancer immunotherapy via dendritic cells. Nat. Rev. Cancer.

[104] Thomas-Kaskel A-K, Waller CF, Schultze-Seemann W, Veelken H (2007). Immunotherapy with dendritic cells for prostate cancer. Int. J. Cancer.

[105] Kantoff PW, Higano CS, Shore ND, Berger ER, Small EJ, Penson DF (2010). Sipuleucel-T immunotherapy for castration-resistant prostate cancer. N. Engl. J. Med..

